# Carrier frequency and incidence of alpha-mannosidosis: population database-based study—focus on the East Asian and Korean population

**DOI:** 10.3389/fgene.2023.1297543

**Published:** 2023-12-01

**Authors:** Jong Eun Park, Taeheon Lee, Kyeongsu Ha, Eun Hye Cho, Chang-Seok Ki

**Affiliations:** ^1^ Department of Laboratory Medicine, Hanyang University Guri Hospital, Hanyang University College of Medicine, Guri, Republic of Korea; ^2^ GC Genome, Yongin, Republic of Korea; ^3^ Department of Laboratory Medicine, Kangbuk Samsung Hospital, Sungkyunkwan University School of Medicine, Seoul, Republic of Korea

**Keywords:** alpha-mannosidosis, *MAN2B1*, carrier frequency, gnomAD, East Asian, Korean

## Abstract

**Background:** Alpha-mannosidosis caused by mutations in the *MAN2B1* gene is a rare genetic disorder characterized by physical abnormalities and intellectual disabilities. The objective of this study was to analyze the carrier frequency and estimated incidence of alpha-mannosidosis in East Asian populations, as limited data exists on its incidence in this group.

**Methods:** In this study, a total of 125,748 exomes from the gnomAD database was analyzed. Additionally, 5,305 data from the KOVA and 1,722 data from the KRGDB, both representing Korean populations, were included.

**Results:** The global carrier frequency of alpha-mannosidosis in gnomAD was 0.23%; the highest carrier frequency was observed in the Finnish at 0.49%, and East Asians had the second highest carrier frequency at 0.30%. Globally, the approximate incidence of alpha-mannosidosis was calculated at 1 in 784,535, l in 166,801 Europeans (Finnish), and l in 431,689 East Asians. By integrating the data from the 8,936 Koreans in gnomAD Korean, KOVA and KRGDB, the carrier frequency of alpha-mannosidosis in the Korean population was 0.04% and estimated incidence was 1 in 19,963,024.

**Conclusion:** This study is the first to investigate the carrier frequencies of alpha-mannosidosis in East Asians and Koreans, including specific subpopulations, utilizing gnomAD and the Korean genomic database. The variant spectrum of *MAN2B1* genes in East Asians showed significant differences compared to other ethnic groups. Our data provide valuable reference information for future investigations into alpha-mannosidosis, aiding in understanding the genetic diversity and specific variants associated with the condition in East Asian populations.

## Introduction

Alpha-mannosidosis is an autosomal recessive lysosomal storage disorder (OMIM #248500) caused by mutations in the *MAN2B1* gene, resulting in reduced activity of the alpha-mannosidase enzyme (Malm and Nilssen, 1993). The deficient activity of alpha-mannosidase leads to a disruption in the breakdown of glycoproteins, causing the gradual buildup of mannose-rich oligosaccharides within lysosomes in all tissues. This accumulation ultimately impairs cellular function and triggers apoptosis (Borgwardt et al., 2014). This rare genetic condition is characterized by facial and skeletal abnormalities, hearing impairment, immune deficiency, and intellectual disability (Malm and Nilssen, 2008). With the recent emergence of enzyme replacement therapy (ERT) for alpha-mannosidase. (Borgwardt et al., 2018; Lund et al., 2018) rapid diagnosis and accurate incidence prediction of alpha-mannosidase are required.

The incidence of alpha-mannosidosis has been reported to range from approximately 1 in 300,000 to 1 in 1,000,000 at birth based on studies conducted among various populations such as Norwegians, Australians, and Czechs (Malm et al., 1995; Meikle et al., 1999; Meikle et al., 2004; Poupetová et al., 2010). However, the incidence of alpha-mannosidosis in East Asians remains relatively unknown, highlighting the need for further investigation and population-specific studies in this region.

Alpha-mannosidosis is a genetically heterogeneous disease where most pathogenic variants are unique to individual families, but some pathogenic variants are repeatedly identified in patients with alpha-mannosidosis. Previous study conducted mainly on European alpha-mannosidosis patients have shown that pathogenic variants c.2248C>T (p.Arg750Trp), c.1830 + 1G>C and c.2426 T>C (p.Leu809Pro) account for 27%, 5%, and 3% of the identified disease alleles, respectively (Riise Stensland et al., 2012). These recurrent variants from alpha-mannosidosis patients provide important insights into the molecular basis of the disorder and can aid in genetic testing and counseling for affected individuals and their families.

The gnomAD (Genome Aggregation Database) is a global genomic database; and gnomAD V2 comprises a vast collection of 125,748 exomes, including 9,197 exomes specifically from East Asian populations (Karczewski et al., 2020). The Korean Variant Archive (KOVA) serves as a reference database for genetic variations in the Korean population, consisting of 1,896 whole genome sequencing data and 3,409 whole exome sequencing data of Koreans (Lee et al., 2022). Another valuable resource is the Korean Reference Genome Database (KRGDB), whole genome sequencing data from 1,722 Koreans (Jung et al., 2020).

Open genome databases are valuable resources as they include genomic data from different ethnic groups, enabling carrier frequency prediction and expected incidence studies. In this study, we analyzed *MAN2B1* variants using exome data from databases interpreted using the 2015 American College of Medical Genetics and Genomics and the Association for Molecular Pathology guidelines (2015 ACMG/AMP guidelines) (Richards et al., 2015). Our study aimed to determine the global carrier frequency and expected incidence of alpha-mannosidosis, with a specific focus on the previously underrepresented East Asian and Korean population.

## Methods

### Population database

We obtained data for the *MAN2B1* gene from gnomAD (v2.1.1) available at https://gnomad.broadinstitute.org/. Our study involved the analysis of 125,748 exomes, which were further categorized into different populations: 9,197 East Asian, 8,128 African/African-American, 17,296 Latino/Admixed American, 5,040 Ashkenazi Jewish, 10,824 Finnish, 56,885 non-Finnish European, 15,308 South Asian, and 3,070 from other populations. Among East Asians, the dataset included 1,909 Koreans, 76 Japanese, and 7,212 individuals from other East Asian backgrounds. Variants flagged with ‘InbreedingCoeff,’ ‘AC0,’ or ‘RF’ QC filters in gnomAD were excluded from our analysis. KOVA and KRGDB were used as Korean databases. KOVA contains data from 5,305 Koreans (https://www.kobic.re.kr/kova/, access date: March 1, 2023), and KRGDB consists of data from 1,722 Koreans (http://coda.nih.go.kr/coda/KRGDB/index.jsp, accessed on 25 September, 2021).

### Interpretation of *MAN2B1* variants and comparative analysis with disease databases

Interpretation of all *MAN2B1* (NM_000528.4) variants followed the 2015 ACMG/AMP guidelines, along with adherence to the Sequence Variant Interpretation general recommendations by ClinGen (https://clinicalgenome.org/working-groups/sequence-variant-interpretation/, accessed on March 10, 2023). These guidelines recommend categorizing variants into five classes: pathogenic variant (PV), likely pathogenic variant (LPV), variants of uncertain significance, likely benign variant, and benign variant. Variant pathogenicity predictions were made using REVEL (Ioannidis et al., 2016) and SpliceAI (Jaganathan et al., 2019). We compared all *MAN2B1* variants identified in population databases with previously classified disease-causing variants from ClinVar and the Human Gene Mutation Database (HGMD), both of which are prominent disease repositories. ClinVar (https://www.ncbi.nlm.nih.gov/clinvar/, accessed on March 2, 2023) offers a freely accessible archive containing variant classifications provided by clinical laboratories. The HGMD professional database (http://www.hgmd.org/, release 2022.04) is a comprehensive repository of germline variants, categorized into six groups, from which we focused solely on disease-causing mutations (DM).

### Alpha-mannosidosis carrier frequency and incidence estimation

Carrier frequencies of alpha-mannosidosis were determined for the *MAN2B1* gene using a population database. We included variants classified as PV and LPV according to the 2015 ACMG-AMP guideline interpretation, DM entries from HGMD, and PV and LPV annotations from ClinVar for the carrier frequency analysis. Subsequently, we estimated the incidence of alpha-mannosidosis based on the calculated frequency and the Hardy–Weinberg equilibrium principle (1 = p^2^ + 2pq + q^2^), where p represents the major allele frequency (non-disease) and q denotes the minor allele frequency (disease). The major allele frequency p was approximated to be close to 1, with 2pq indicating carrier frequency and q^2^ signifying the disease state. We estimated carrier frequencies using the sum of alleles interpreted as PV/LPV based on the population for which exome sequencing was performed. This allowed us to calculate the carrier frequency and subsequently derive the q value. Ultimately, we used the q value to predict q^2^ to estimate disease incidence. We conducted statistical analysis using MedCalc ver. 11.5.1.0 (MedCalc Software, Maiakerke, Belgium), and we computed 95% confidence intervals for carrier frequency and estimated disease incidence.

## Results

**FIGURE 1:**
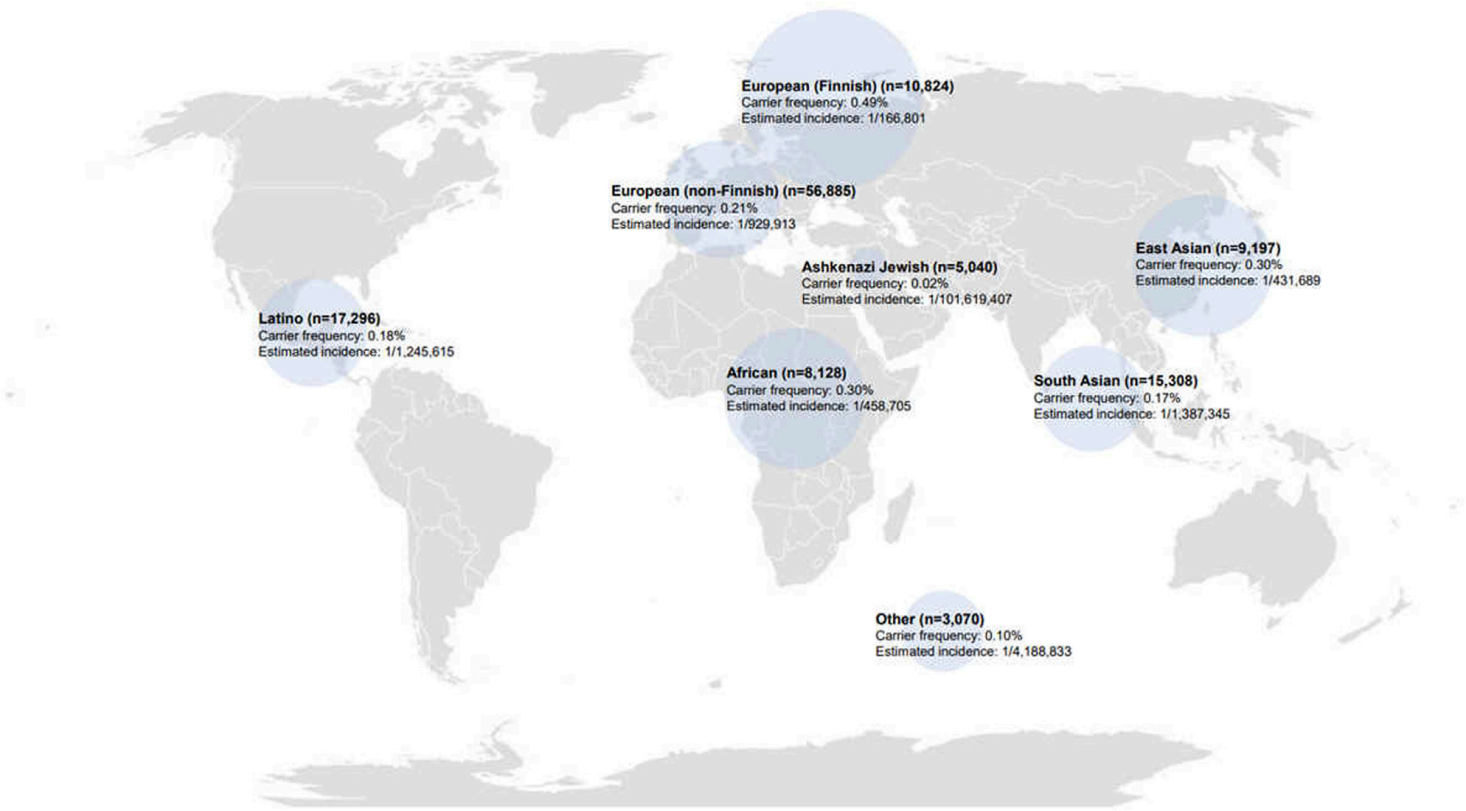
Carrier frequency and estimated incidence of alpha-mannosidosis in gnomAD according to population. Circle size: carrier frequency of alpha-mannosidosis.

We conducted an analysis of 125,748 exomes, which included 9,197 East Asian exomes from the gnomAD database, focusing on variants within the *MAN2B1* gene. Variant classification followed the 2015 ACMG/AMP guidelines, and we additionally compared these variants with data from two disease classification databases, HGMD and ClinVar (Table 1). Based on the 2015 ACMG/AMP guidelines, the global carrier frequency of alpha-mannosidosis was reported to be 0.23%. The carrier frequency and estimated incidence for each population are as follows (Figure 1): Among populations, the highest carrier frequency was observed in European (Finnish) at 0.49%. East Asians had the second highest carrier frequency at 0.30%. In contrast, the lowest carrier frequency was found among Ashkenazi Jewsh, 0.02%. The estimated incidence of alpha-mannosidosis was 1 in 784,535 worldwide, l in 166,801 European (Finnish) and l in 431,689 East Asians. According to ClinVar, the global carrier frequency for alpha-mannosidosis was reported as 0.16%, resulting in an estimated incidence of 1 in 1,505,514. HGMD data suggests a global carrier frequency of 0.67% for alpha-mannosidosis, resulting in an estimated incidence of 1 in 90,289.

**TABLE 1:** Carrier frequency and estimated incidence of alpha-mannosidosis in gnomAD.

	Total alleles (n)	Carrier frequency (%), (95% CI)	Estimated incidence (1/n), (95% CI)
Total (*n* = 125,748)			
ACMG (PV/LPV)	284	0.23 (0.20–0.25)	1/784,535 (1/997,007–1/621,468)
ClinVar (PV/LPV)	205	0.16 (0.14–0.19)	1/1,505,514 (1/1,997,777–1/1,145,095)
HGMD (DM)	837	0.67 (0.62–0.71)	1/90,289 (1/103,623–1/78,838)
East Asian (n = 9,197)			
ACMG (PV/LPV)	28	0.30 (0.20–0.44)	1/431,689 (1/977,391–1/206,612)
ClinVar (PV/LPV)	3	0.03 (0.01–0.10)	1/37,591,710 (1/883,140,625–1/4,401,501)
HGMD (DM)	4	0.04 (0.01–0.11)	1/21,148,578 (1/284,854,635–1/3,225,533)
African (n = 8,128)			
ACMG (PV/LPV)	24	0.30 (0.19–0.44)	1/458,705 (1/1,117,423–1/207,271)
ClinVar (PV/LPV)	22	0.27 (0.17–0.41)	1/545,863 (1/1,390,619–1/238,186)
HGMD (DM)	17	0.21 (0.12–0.33)	1/913,980 (1/2,696,283–1/356,640)
Latino (n = 17,296)			
ACMG (PV/LPV)	31	0.18 (0.12–0.25)	1/1,245,615 (1/2,696,283–1/618,053)
ClinVar (PV/LPV)	6	0.03 (0.01–0.08)	1/33,239,263 (1/246,832,979–1/7,015,378)
HGMD (DM)	134	0.77 (0.65–0.92)	1/66,649 (1/94,937–1/47,507)
Ashkenazi Jewish (n = 5,040)			
ACMG (PV/LPV)	1	0.02 (0.00–0.11)	1/101,619,407 (1/160,000,000,000–1/3,272,974)
ClinVar (PV/LPV)	0	0 (0–0.07)	1/NA (1/NA - 1/8,163,265)
HGMD (DM)	14	0.28 (0.15–0.47)	1/518,317 (1/1,733,582–1/184,120)
European (Finnish) (n = 10,824)			
ACMG (PV/LPV)	53	0.49 (0.37–0.64)	1/166,801 (1/297,304–1/97,504)
ClinVar (PV/LPV)	53	0.49 (0.37–0.64)	1/166,801 (1/297,304–1/97,504)
HGMD (DM)	125	1.16 (0.96–1.38)	1/29,984 (1/43,312–1/21,126)
European (non-Finnish) (n = 56,885)			
ACMG (PV/LPV)	118	0.21 (0.17–0.25)	1/929,913 (1/1,356,811–1/648,271)
ClinVar (PV/LPV)	102	0.18 (0.15–0.22)	1/1,244,226 (1/1,871,394–1/844,001)
HGMD (DM)	498	0.88 (0.80–0.96)	1/52,185 (1/62,469–1/43,785)
South Asian (n = 15,308)			
ACMG (PV/LPV)	26	0.17 (0.11–0.25)	1/1,387,345 (1/3,252,347–1/645,669)
ClinVar (PV/LPV)	17	0.11 (0.06–0.18)	1/3,240,648 (1/9,555,456–1/1,265,309)
HGMD (DM)	13	0.08 (0.05–0.15)	1/5,546,768 (1/19,561,352–1/1,896,737)
Other (n = 3,070)			
ACMG (PV/LPV)	3	0.10 (0.02–0.29)	1/4,188,833 (1/98,516,708–1/490,461)
ClinVar (PV/LPV)	2	0.07 (0.01–0.24)	1/9,423,910 (1/642,548,605–1/722,280)
HGMD (DM)	32	1.04 (0.71–1.47)	1/36,840 (1/78,683–1/18,486)

2015 ACMG/AMP, 2015 American College of Medical Genetics and Genomics and the Association for Molecular Pathology guideline; *95% CI*, 95% confidence intervals; *DM*, disease-causing variant; *gnomAD*, genome aggregation database; *LPV*, likely pathogenic variant; *NA*, not applicable; *PV*, pathogenic variant.

According to 2015 ACMG/AMP guidelines, among East Asian populations in the gnomAD database, no PV/LPVs associated with alpha-mannosidosis were found in Koreans or Japanese (Supplementary Table S1). Therefore, the carrier frequency for alpha-mannosidosis in both populations was 0% in gnomAD. As a result, we could not evaluate the estimated incidence of alpha-mannosidosis in these specific East Asian populations based on the available data. The carrier frequency in other East Asian populations in gnomAD was 0.39%, and the estimated incidence was 1 in 265,372.

Based on the analysis conducted in accordance with the 2015 ACMG/AMP guidelines, the carrier frequency of alpha-mannosidosis in the Korean population database KOVA (n = 5,305) was determined to be 0.06%. Similarly, in the KRGDB database (n = 1,722), the carrier frequency was also 0.06%. The estimated incidence of alpha-mannosidosis in these populations was calculated to be 1 in 12,508,011 for the KOVA data and 1 in 11,861,136 for the KRGDB data (Supplementary Table S1). By integrating the data from the 8,936 individuals in gnomAD Korean, KOVA and KRGDB, the carrier frequency of alpha-mannosidosis in the Korean population was 0.04% and estimated incidence was 1 in 19,963,024.

Table 2 summarizes the PVs/LPVs in the *MAN2B1* gene found in the gnomAD database. A total of 81 different variants were identified, comprising 284 alleles. Among them, 30 were missense variants (175 alleles), 22 were nonsense variants (48 alleles), 16 were frameshift variants (17 alleles), and 12 were splicing variants (43 alleles). Additionally, one synonymous variant was identified (1 allele). The most common variant worldwide was c.2248C>T, p.(Arg750Trp), with 59 alleles identified. This variant was frequently observed across multiple ethnicities but particularly in the European (Finnish) population. Following the most frequent variant, the next most common variants observed were c.565C>A, p.(Pro189Thr), and c.1830 + 1G>C, each found in 23 alleles. The c.565C>A variant was exclusively detected in Europeans (Finnish and non-Finnish), while the c.1830 + 1G>C variant was found only in East Asians. Additional analysis comparing PVs/LPVs in East Asians with those in other ethnic groups revealed significant distinctions. The PVs/LPVs found in East Asian populations were absent in Latino, Ashkenazi Jewish, and South Asian populations.

**TABLE 2:** Pathogenic variants and likely pathogenic variants in gnomAD.

Nucleotide change	Amino acid change	gnomAD allele frequency								
		Total	East Asain	African	Latino	Ashkenazi Jewish	European (Finnish)	European (non-Finnish)	South Asian	Other
		(*n* = 125,748)	(*n* = 9,197)	(*n* = 8,128)	(*n* = 17,296)	(*n* = 5,040)	(*n* = 10,824)	(*n* = 56,885)	(*n* = 15,308)	(*n* = 3,070)
c.12C>G	p.(Tyr4*)	3.98.E-06	0	0	0	0	0	0	4.41.E-05	0
c.65G>A	p.(Trp22*)	7.95.E-06	0	2.71.E-04	0	0	0	0	0	0
c.159 + 2T>A	p.?	3.98.E-06	0	0	0	0	0	0	0	2.03.E-04
c.175C>T	p.(Gln59*)	3.98.E-06	0	0	0	0	0	0	3.27.E-05	0
c.222C>A	p.(Asp74Glu)	1.99.E-05	0	0	0	0	0	4.39.E-05	0	0
c.233T>C	p.(Leu78Pro)	3.98.E-06	0	0	0	0	0	8.79.E-06	0	0
c.241del	p.(Val81Trpfs*76)	3.98.E-06	0	0	0	0	0	8.79.E-06	0	0
c.308C>T	p.(Ser103Leu)	3.98.E-06	0	0	0	0	0	0	3.27.E-05	0
c.308del	p.(Ser103Trpfs*54)	3.98.E-06	0	0	0	0	0	8.79.E-06	0	0
c.388C>T	p.(Gln130*)	3.98.E-06	0	0	2.89.E-05	0	0	0	0	0
c.418C>T	p.(Arg140*)	1.59.E-05	0	0	2.89.E-05	0	0	1.76.E-05	3.27.E-05	0
c.422del	p.(Asp141Alafs*16)	3.99.E-06	0	0	0	0	0	8.82.E-06	0	0
c.437-1G>A	p.?	3.99.E-06	0	6.17.E-05	0	0	0	0	0	0
c.438dup	p.(Arg147Alafs*14)	3.99.E-06	0	0	0	0	0	8.83.E-06	0	0
c.458G>T	p.(Gly153Val)	3.98.E-06	0	0	2.89.E-05	0	0	0	0	0
c.565C>A	p.(Pro189Thr)	9.15.E-05	1.25.E-03	0	0	0	0	0	0	0
c.566C>A	p.(Pro189His)	3.98.E-06	0	0	0	0	0	8.84.E-06	0	0
c.589C>T	p.(Pro197Ser)	3.98.E-06	0	0	0	0	0	0	3.27.E-05	0
c.595G>A	p.(Gly199Ser)	3.98.E-06	0	0	0	0	0	8.88.E-06	0	0
c.598C>A	p.(His200Asn)	7.97.E-06	0	0	2.90.E-05	0	0	8.89.E-06	0	0
c.630 + 1G>C	p.?	3.98.E-06	0	6.27.E-05	0	0	0	0	0	0
c.643G>A	p.(Gly215Ser)	3.98.E-06	0	0	0	9.92.E-05	0	0	0	0
c.644G>T	p.(Gly215Val)	3.98.E-06	0	0	0	0	0	8.79.E-06	0	0
c.680G>A	p.(Trp227*)	3.98.E-06	5.44.E-05	0	0	0	0	0	0	0
c.685C>T	p.(Arg229Trp)	1.59.E-05	5.44.E-05	6.15.E-05	0	0	4.62.E-05	8.79.E-06	0	0
c.788C>T	p.(Pro263Leu)	7.95.E-06	0	0	2.89.E-05	0	4.63.E-05	0	0	0
c.807G>A	p.(Trp269*)	3.98.E-06	0	0	0	0	0	0	3.27.E-05	0
c.856G>A	p.(Glu286Lys)	1.19.E-05	0	0	2.89.E-05	0	0	1.76.E-05	0	0
c.861C>A	p.(Tyr287*)	3.98.E-06	0	0	2.89.E-05	0	0	0	0	0
c.956A>G	p.(Asp319Gly)	3.98.E-06	0	0	2.89.E-05	0	0	0	0	0
c.1026 + 2T>G	p.?	1.59.E-05	0	0	0	0	0	3.52.E-05	0	0
c.1064C>T	p.(Thr355Ile)	3.98.E-06	0	0	0	0	0	8.80.E-06	0	0
c.1117A>T	p.(Lys373*)	3.98.E-06	0	6.15.E-05	0	0	0	0	0	0
c.1135C>T	p.(Pro379Ser)	3.58.E-05	0	0	0	0	0	0	2.94.E-04	0
c.1140C>A	p.(Tyr380*)	3.98.E-06	0	0	0	0	0	8.79.E-06	0	0
c.1169G>C	p.(Gly390Ala)	8.75.E-05	0	0	6.36.E-04	0	0	0	0	0
c.1321_1327del	p.(Ala441Serfs*34)	3.98.E-06	7.62.E-05	0	0	0	0	0	0	0
c.1383C>A	p.(Tyr461*)	1.19.E-05	0	0	0	0	0	4.38.E-05	0	0
c.1501T>A	p.(Cys501Ser)	3.98.E-06	0	0	0	0	0	8.84.E-06	0	0
c.1572G>A	p.(Trp524*)	3.98.E-06	0	0	0	0	0	8.79.E-06	0	0
c.1645-1G>A	p.?	7.95.E-06	0	0	0	0	0	1.77.E-05	0	0
c.1687G>T	p.(Glu563*)	3.98.E-06	0	0	2.89.E-05	0	0	0	0	0
c.1830 + 1G>C	p.?	9.15.E-05	0	0	0	0	3.26.E-04	1.42.E-04	0	0
c.1858dup	p.(Thr620Asnfs*31)	3.98.E-06	0	0	0	0	0	8.80.E-06	0	0
c.1859_1860del	p.(Thr620Argfs*30)	3.98.E-06	0	6.15.E-05	0	0	0	0	0	0
c.1894C>T	p.(Gln632*)	3.98.E-06	0	0	0	0	0	0	3.27.E-05	0
c.1929-1G>T	p.?	7.95.E-06	0	1.23.E-04	0	0	0	0	0	0
c.1963del	p.(Gln655Argfs*18)	3.98.E-06	0	0	0	0	0	8.79.E-06	0	0
c.2046 + 2T>A	p.?	1.99.E-05	0	3.08.E-04	0	0	0	0	0	0
c.2088G>A	p.(Trp696*)	3.98.E-06	0	0	0	0	4.64.E-05	0	0	0
c.2175G>A	p.(Trp725*)	3.98.E-06	0	0	0	0	0	8.79.E-06	0	0
c.2237_2240del	p.(Asp746Alafs*19)	3.98.E-06	0	0	0	0	0	0	3.27.E-05	0
c.2246G>A	p.(Gly749Asp)	3.98.E-06	0	0	0	0	0	8.79.E-06	0	0
c.2248C>T	p.(Arg750Trp)	2.35.E-04	5.44.E-05	1.85.E-04	0	0	1.11.E-03	2.64.E-04	0	1.63.E-04
c.2249G>A	p.(Arg750Gln)	1.19.E-05	0	1.23.E-04	0	0	0	8.79.E-06	0	0
c.2278C>T	p.(Arg760*)	1.19.E-05	0	1.25.E-04	0	0	0	0	3.29.E-05	0
c.2299C>T	p.(Gln767*)	7.55.E-05	0	0	0	0	8.85.E-04	0	0	0
c.2345_2348dup	p.(Ile784Leufs*14)	3.99.E-06	0	6.17.E-05	0	0	0	0	0	0
c.2355G>A	p.(Thr785 = )	3.99.E-06	0	0	0	0	0	8.82.E-06	0	0
c.2355 + 1G>C	p.?	3.99.E-06	0	0	0	0	0	8.82.E-06	0	0
c.2373del	p.(Thr792Leufs*3)	3.98.E-06	0	0	0	0	0	8.79.E-06	0	0
c.2398G>A	p.(Gly800Arg)	3.98.E-06	0	0	0	0	0	8.79.E-06	0	0
c.2398G>C	p.(Gly800Arg)	7.95.E-06	5.44.E-05	0	0	0	0	8.79.E-06	0	0
c.2398G>T	p.(Gly800Trp)	3.98.E-06	0	6.15.E-05	0	0	0	0	0	0
c.2401G>A	p.(Gly801Ser)	3.98.E-06	0	0	0	0	0	0	3.27.E-05	0
c.2402del	p.(Gly801Alafs*4)	3.98.E-06	0	0	0	0	0	8.80.E-06	0	0
c.2414_2417del	p.(Arg805Metfs*13)	3.98.E-06	0	0	0	0	0	8.80.E-06	0	0
c.2426T>C	p.(Leu809Pro)	6.76.E-05	0	0	0	0	0	1.50.E-04	0	0
c.2436 + 1G>A	p.?	3.98.E-06	0	0	0	0	0	8.82.E-06	0	0
c.2437-2A>G	p.?	3.98.E-06	0	0	0	0	0	9.36.E-06	0	0
c.2581G>T	p.(Glu861*)	3.98.E-06	0	7.39.E-05	0	0	0	0	0	0
c.2696C>A	p.(Ser899*)	3.98.E-06	0	0	0	0	0	9.00.E-06	0	0
c.2723G>A	p.(Trp908*)	3.98.E-06	0	0	0	0	0	8.92.E-06	0	0
c.2724G>A	p.(Trp908*)	4.04.E-06	0	0	0	0	0	8.92.E-06	0	0
c.2747G>A	p.(Arg916His)	7.95.E-06	0	0	0	0	0	8.85.E-06	0	1.63.E-04
c.2747G>T	p.(Arg916Leu)	3.98.E-06	0	0	0	0	0	8.85.E-06	0	0
c.2804_2811del	p.(Val935Glufs*?)	7.95.E-06	0	0	0	0	0	1.76.E-05	0	0
c.2821-1G>A	p.?	3.98.E-06	0	0	0	0	0	0	3.27.E-05	0
c.2867T>G	p.(Leu956Arg)	2.39.E-05	0	0	0	0	0	0	1.96.E-04	0
c.2869del	p.(Val957Trpfs*76)	3.98.E-06	0	0	0	0	0	8.79.E-06	0	0
c.2821-42_2872dup	p.(Ala958Glyfs*?)	3.98.E-06	0	0	0	0	0	8.79.E-06	0	0

*gnomAD*, genome aggregation database.

## Discussion

Within this study, the carrier frequency and estimated incidence of alpha-mannosidosis were analyzed using gnomAD and the Korean database. According to our evaluation of gnomAD data, the global carrier frequency of alpha-mannosidosis was established at 0.23%, whereas in East Asians, it was slightly higher at 0.30%. Ashkenazi Jewish individuals exhibited the lowest carrier frequency, which was recorded at 0.02%. Carrier frequency studies of alpha-mannosidosis are rare, which poses challenges in directly comparing our findings with existing literature. However, considering the reported incidence rates of alpha-mannosidosis from previous studies, which range from approximately 1 in 300,000 to 1 in 1,000,000 (Malm et al., 1995; Meikle et al., 1999; Meikle et al., 2004; Poupetová et al., 2010), our estimated incidence of 1 in 784,535 is consistent.

The carrier frequency and incidence rates of alpha-mannosidosis in East Asian populations were examined for the first time in this study. Our findings reveal high carrier frequency of alpha-mannosidosis in East Asians (0.30%). However, our study also reveals that alpha-mannosidosis is significantly rare among Koreans, one of the major East Asian groups. By incorporating data from the KOVA and KRGDB databases, our analysis of 8,936 Koreans showed a carrier frequency of 0.04%. This carrier frequency is as low as the lowest frequency observed in Ashkenazi Jewish (0.02%) in the gnomAD. This highlights a distinct pattern in the carrier frequency and estimated incidence of alpha-mannosidosis among ethnic groups and emphasizes the need for population-specific studies to better understand the disease’s distribution and genetic factors contributing to its occurrence.

Based on data obtained from the Korean Statistical Information Service (http://kosis.kr/; accessed on June 20, 2023), as of 2021, Korea had a total population of 51.7 million and recorded 260,562 births. Considering the carrier frequency determined in this study, it is estimated that there are approximately 21,000 carriers in the entire population and 104 carriers among newborns each year. The estimated incidence of alpha-mannosidosis in Korea, calculated using the Hardy–Weinberg equilibrium, is approximately 0.01 cases per year. Thus, the expected number of eligible patients for enzyme replacement therapy (ERT) in Korea is likely to be quite small.

The ACMG practice resource categorizes conditions into tiers based on carrier frequency for carrier screening during pregnancy and preconception (Gregg et al., 2021). Alpha-mannosidosis is not included in the Tier 3 genes recommended for screening. However, the BabySeq project, a randomized controlled trial for genetic newborn screening, classifies the *MAN2B1* gene as Category A, emphasizing its significance in newborn screening for highly penetrant childhood-onset disorders (Ceyhan-Birsoy et al., 2017). The rarity and variable severity of alpha-mannosidosis can lead to a diagnostic delay of over a decade (Guffon et al., 2019). Early diagnosis is crucial for optimal treatment outcomes, including interventions such as allogeneic hematopoietic stem cell transplantation or ERT (Mynarek et al., 2012; Borgwardt et al., 2018). Recognizing the importance of early detection and intervention is essential for the wellbeing of at-risk infants. Notably, our study revealed that, among East Asians, the carrier frequency is the second-highest following the European (Finnish) population. This highlights the importance of active genetic screening in the East Asian region as it is likely to contribute to the identification and treatment of affected individuals.

Among the PVs/LPVs in the *MAN2B1* gene identified in the gnomAD database, the most prevalent variant worldwide was c.2248C>T, p.(Arg750Trp). This finding aligns with previous studies indicating this as the most frequently observed variant among alpha-mannosidosis patients (Riise Stensland et al., 2012). The c.2248C>T, p.(Arg750Trp) variant is common in European (Finnish) populations but has been observed in other ethnic groups, including East Asian and African populations. This indicates that the variant is not limited to a specific racial or ethnic background. The next most frequently observed variants were c.565C>A, p.(Pro189Thr) and c.1830 + 1G>C. Among these, the c.1830 + 1G>C variant was the second most common variant found in alpha-mannosidosis patients (Riise Stensland et al., 2012). The c.565C>A, p.(Pro189Thr) variant is specifically observed in East Asian populations and has not been reported in other ethnic groups. Since previous studies mainly included Western alpha-mannosidosis patients, the results indicate a significant concordance between the variants identified in population databases and those found in actual cases of alpha-mannosidosis. This highlights the utility of population databases as valuable resources for studying the prevalence and distribution of specific mutations in different populations.

This study has several limitations. Firstly, this study did not assess structural variations, such as large deletions or insertions in the *MAN2B1* gene, which may have led to an underestimation of the carrier frequency of alpha-mannosidosis. While reported cases of alpha-mannosidosis resulting from structural mutations in the *MAN2B1* gene are limited (Riise Stensland et al., 2012; Wiesinger et al., 2020), this is likely to have had a minimal impact on the underestimation of carrier frequency. Second, since the estimated incidence of alpha-mannosidosis in this study was derived entirely from genetic information, the accuracy of the estimated incidence may be impaired. Lastly, in gnomAD, for subpopulations with smaller sample sizes such as Koreans and Japanese, there were instances where PV/LPV were not identified. As a result, this has led to limitations in accurate assessment and estimation of carrier frequency.

Nevertheless, this study effectively verified the carrier frequency and estimated incidence of alpha-mannosidosis across ethnicities. Moreover, this is the largest study in East Asians, including Koreans, to analyze the *MAN2B1* gene. To our knowledge, there is a lack of large-scale population studies on the carrier frequency and estimated incidence of alpha-mannosidosis, specifically in Koreans. Therefore, we believe that this study provides a more accurate prediction of the carrier frequency in both East Asians and Koreans. Given the recent advancements in treatments for alpha-mannosidosis, identifying the carrier frequency and incidence for effective management and intervention is increasingly important.

## Conclusion

This study is the first to investigate the carrier frequencies of alpha-mannosidosis in East Asians, including Koreans, utilizing gnomAD and the Korean genomic database. Our findings confirmed that, among various ethnic groups, East Asians are second in terms of carrier frequency of alpha-mannosidosis. However, among East Asians, Koreans exhibited significantly lower carrier frequencies. This implies that Koreans have a notably lower prevalence of alpha-mannosidosis than other populations, including other East Asian populations. The variant spectrum of *MAN2B1* genes in East Asians showed significant differences compared to other ethnic groups. Our data provide valuable reference information for future investigations into alpha-mannosidosis, aiding in understanding the genetic diversity and specific variants associated with the condition in East Asian populations.

## Data Availability

The original contributions presented in the study are included in the article/Supplementary Material, further inquiries can be directed to the corresponding author.
