# Supplementary material for: Carrier frequency and incidence of alpha-mannosidosis: population database-based study—focus on the East Asian and Korean population
Source: Front Genet. 2023 Dec 1;14:1297543. doi: 10.3389/fgene.2023.1297543 (PMC10725197; doi:10.3389/fgene.2023.1297543)
Supplement: Supplementary file 1 [file Table1.docx]

**Supplementary Table 1.** Carrier frequency and estimated incidence of alpha-mannosidosis in East Asian and Korean

|  | Total alleles (n) | Carrier frequency (%), (95% CI) | Estimated incidence (1/n), (95% CI) |
| --- | --- | --- | --- |
| gnomAD East Asian exomes (n=9,197) |  |  |  |
| ACMG (PV/LPV) | 28 | 0.30 (0.20 - 0.44) | 1/431,689 (1/977,391 - 1/206,612) |
| ClinVar (PV/LPV) | 3 | 0.03 (0.01 - 0.10) | 1/37,591,710 (1/883,140,625 - 1/4,401,501) |
| HGMD (DM) | 4 | 0.04 (0.01 - 0.11) | 1/21,148,578 (1/284,854,635 - 1/3,225,533) |
| gnomAD Korean exomes (n=1,909) |  |  |  |
| ACMG (PV/LPV) | 0 | 0.00 (0.00 - 0.19) | NA (NA - 1/1,108,033) |
| ClinVar (PV/LPV) | 0 | 0.00 (0.00 - 0.19) | NA (NA - 1/1,108,033) |
| HGMD (DM) | 0 | 0.00 (0.00 - 0.19) | NA (NA - 1/1,108,033) |
| gnomAD Japanese exomes (n=76) |  |  |  |
| ACMG (PV/LPV) | 0 | 0 (0 - 4.85) | NA (NA - 1/1,700) |
| ClinVar (PV/LPV) | 0 | 0 (0 - 4.85) | NA (NA - 1/1,700) |
| HGMD (DM) | 0 | 0 (0 - 4.85) | NA (NA - 1/1,700) |
| gnomAD Other East Asian exomes (n=7,212) |  |  |  |
| ACMG (PV/LPV) | 28 | 0.39 (0.26 - 0.56) | 1/265,372 (1/600,925 - 1/127,051) |
| ClinVar (PV/LPV) | 3 | 0.04 (0.01 - 0.12) | 1/23,116,864 (1/543,357,187 - 1/2,706,495) |
| HGMD (DM) | 4 | 0.06 (0.02 - 0.14) | 1/13,003,236 (1/175,198,774 - 1/1,983,454) |
| All Korean (n=8,936) |  |  |  |
| ACMG (PV/LPV) | 4 | 0.04 (0.01 - 0.11) | 1/19,963,024 (1/268,744,961 - 1/3,045,194) |
| ClinVar (PV/LPV) | 2 | 0.02 (0.00 - 0.08) | 1/79,852,096 (1/5,446,548,930 - 1/6,119,275) |
| HGMD (DM) | 1 | 0.01 (0.00 - 0.06) | 1/319,408,384 (1/510,204,081,633 - 1/10,289,330) |
| gnomAD Korean exomes (n=1,909) |  |  |  |
| ACMG (PV/LPV) | 0 | 0.00 (0.00 - 0.19) | NA (NA - 1/1,108,033) |
| ClinVar (PV/LPV) | 0 | 0.00 (0.00 - 0.19) | NA (NA - 1/1,108,033) |
| HGMD (DM) | 0 | 0.00 (0.00 - 0.19) | NA (NA - 1/1,108,033) |
| KOVA (n=5,305) |  |  |  |
| ACMG (PV/LPV) | 3 | 0.06 (0.01 - 0.17) | 1/12,508,011 (1/294,213,699 - 1/1,464,618) |
| ClinVar (PV/LPV) | 1 | 0.02 (0.00 - 0.11) | 1/112,572,100 (1/173,611,111,111 - 1/3,626,046) |
| HGMD (DM) | 1 | 0.02 (0.00 - 0.11) | 1/112,572,100 (1/173,611,111,111 - 1/3,626,046) |
| KRGDB (n=1,722) |  |  |  |
| ACMG (PV/LPV) | 1 | 0.06 (0.00 - 0.32) | 1/11,861,136 (1/18,510,805,683 - 1/382,077) |
| ClinVar (PV/LPV) | 1 | 0.06 (0.00 - 0.32) | 1/11,861,136 (1/18,510,805,683 - 1/382,077) |
| HGMD (DM) | 0 | 0.00 (0.00 - 0.21) | NA (NA - 1/907,029) |

*2015 ACMG/AMP*, 2015 American College of Medical Genetics and Genomics and the Association for Molecular Pathology guideline; *95% CI*, 95% confidence intervals; *DM*, disease-causing variant; *gnomAD*, Genome Aggregation Database; *LPV*, likely pathogenic variant; *NA*, not applicable; *PV*, pathogenic variant.
